# Integrating County-Level Socioeconomic Data for COVID-19 Forecasting in the United States

**DOI:** 10.1109/OJEMB.2021.3096135

**Published:** 2021-07-09

**Authors:** Michael C. Lucic, Hakim Ghazzai, Carlo Lipizzi, Yehia Massoud

**Affiliations:** Stevens Institute of Technology 33694 Hoboken NJ 07030 USA; Computer, Electrical and Mathematical Sciences and Engineering DivisionKing Abdullah University of Science and Technology 127355 Thuwal 23955-6900 Saudi Arabia

**Keywords:** ARIMA, COVID-19, data analytics, $k$-means clustering, time series analysis

## Abstract

*Goal:* The United States (US) is currently one of the countries hardest-hit by the novel SARS-CoV-19 virus. One key difficulty in managing the outbreak at the national level is that due to the US’ diversity, geographic spread, and economic inequality, the COVID-19 pandemic in the US acts more as a series of diverse regional outbreaks rather than a synchronized homogeneous one. *Method:* In order to determine how to assess regional risk related to COVID-19, a two-phase modeling approach is developed while considering demographic and economic criteria. First, an unsupervised clustering technique, specifically $k$-means, is employed to group US counties based on demographic and economic similarities. Then, time series forecasting of each cluster of counties is developed to assess the short-run viral transmissibility risk. *Results:* To this end, we test ARIMA and Seasonal Trend Random Walk forecasts to determine which is more appropriate for modeling the spread and lethality of COVID-19. From our analysis, we then utilize the superior ARIMA models to forecast future COVID-19 trends in the clusters, and present the areas in the US which have the highest COVID-19 related risk heading into the winter of 2020. *Conclusion:* Including sub-national socioeconomic characteristics to data-driven COVID-19 infection and fatality forecasts may play a key role in assessing the risk associated with changes in infection patterns at the national level.

## I. Introduction

Throughout 2020, the novel SARS-CoV-2 virus driving the COVID-19 pandemic has completely changed the world, and has particularly affected the United States (US). In a one-year period starting with the detection of community spread in late February 2020, the virus infected almost 28 million Americans and is confirmed to have led to over 425,000 fatalities as a result of various complications from infection [1].

There have been numerous factors that have complicated the American federal-level response to COVID-19. These include medical considerations, such as the ability of the virus to ride along aerosolized particles that an infected individual breathes out [2], and political ones [3]. Less-discussed is the vast geographic spread of the US. Notably, the series of infection waves in the US have led to surges of cased that were more concentrated in certain regions of the US. The appearance of a series of loosely-connected regional outbreaks versus a coordinated set of waves of infection that appear all at once nationally is partially the result of the large geographical and demographic diversity in the US. When considering this regional-outbreak perspective, it may be prudent to segment sections of the country into distinct groups. At a population-level, groups of people with similar demographics and/or socioeconomic standing are likely to have similar behaviors due to cultural similarities. It is therefore worthwhile to build viral case forecasting models that take this into account.

Since COVID-19 began to spread globally in Spring 2020, researchers have applied various mathematical models with the intent to forecast future COVID-19 cases. According to the authors of [4], models that are utilized in epidemiological settings tend to either be mechanistic models based on grounded epidemiological theory or physical system characteristics, or are data-driven statistical models. Mechanistic models aim to leverage both the infection/fatality data in a pandemic and known physical processes that underpin the spread of a virus in order to model the dynamic change in the number of infected people as time progresses [5]. Examples of less complex mechanistic models include the Susceptible-Infected-Recovered (SIR) models [6], [7] and the Susceptible-Exposed-Infectious-Recovered (SEIR) models [8]. These models are effective at developing quick insights into how a pandemic may progress, and may include varying degrees of complexity, based on the approach of the developers [9]. Subsequently, they have been applied to model the progression of the COVID-19 pandemic in China [10] and Canada [11]. Due to the lower degree of complexity of these models, as they only model three or four potential system states, there have been attempts to apply or extend them in applications related to COVID-19. The authors of [12] and [13] developed generalized mechanistic models that consider seven and eight (respectively) potential states the population may exist in as a result of COVID-19: susceptible, protected (quarantined), exposed, infected, infected and isolated, hospitalized (only in [13]), dead, and recovered. They found that their approach improved performance versus Susceptible-Exposed-Infectious-Recovered-Dead (SEIRD) models. Another approach that aimed to apply mechanistic approaches to model infection waves through “Riccati Modules” at the national-level in the US was developed by the author of [14].

In the context of epidemiological modeling, data-driven models aim to model the progression of the virus over time while not considering epidemiological-specific characteristics like in mechanistic models. The Generalized Growth Model (GGM) and Generalized Richards Model (GRM) [15]–[19] are commonly used data-driven approaches that aim to fit spread trends to a generalized logistic curve. While these models can provide an outlook for the entire progression of a virus, they are not as effective as mechanistic models at this. In addition, they are somewhat rigid, preventing the consideration of rapid changes on a day-to-day or week-to-week basis as the result of public health policy or virus mutations. Other data-driven approaches that utilize time series models such as ARIMA [20]–[24], or deep learning models such as LSTM [25]–[27] can be leveraged to adapt as the circumstances change. ARIMA models tend to be more prevalent in medical applications, due to the ease in building explainable models versus deep learning approaches. Due to the serious nature of the problem being explored in this study, as well as the constantly changing circumstances, ARIMA models may be a more appropriate selection, as they can be rapidly refit as more data becomes available, and the relationships may be more clearly explained.

Oftentimes, the application of models in epidemiological studies must consider factors that may lead to variations in the transmissibility of a disease – one population group may exhibit different behaviors than others due to various sociological and cultural factors, which may result in different population-level viral spread. While the aforementioned models are effective for modeling the progression of a pandemic for a region, we must consider multiple regions. As these regions may supersede state boundaries in the US, counties may be considered as the “building blocks” for regions based on socioeconomic characteristics. Consequently, clustering approaches are appropriate for just this task. There are various areas that leverage the use of clustering algorithms such as $k$-means clustering [28] to partition large population-level datasets into clusters that are grouped together based on similarity. Clustering approaches are commonly applied to demographic features in various applications for grouping users/subjects based on similarity. These include recommendation systems [29], sociological analysis [30], data mining for inductive analysis [31], and for the development of policy [32].

For the work presented in this paper, we have gathered several county-level datasets pertaining to the US. These datasets include information on population demographics, property development, financial productivity, and daily cumulative case counts and fatalities as a result of COVID-19. From this data, we plan to leverage demographic and socioeconomic data to group American counties together into large aggregations based on their similarities to one another. These aggregate groups of counties that are similar should have similar cumulative case count and fatality data, allowing for us to develop case and fatality forecasts for each to assess of how COVID-19 is expected to continue spreading if measures continue as-is. Based on this, we aim to develop a two-stage forecasting system. After organizing the data into a tidy form, we will first apply unsupervised learning approaches such as $k$-means clustering to group the counties together based on their socioeconomic profiles. Subsequently, we will aggregate the time series for each county together based on its assigned cluster to develop cluster-level time series forecasts. From our analysis, clustering with $k$-means and time series forecasting with Autoregressive Integrated Moving Average (ARIMA) leads to the most effective fits and forecasts. We then aggregate the cluster-level ARIMA fits and forecasts to develop a national-level forecast, and see that it comes close to matching the performance of a dedicated national-level ARIMA forecast.

The aim of these models is to identify high-risk areas where public health measures should be focused. To our knowledge, there does not exist a system that leverages a combination of clustering on demographic data and data-driven models in order to forecast COVID-19 progression across a country. The vast majority of the existing literature related to modeling COVID-19 seems to focus on producing national-level forecasts. The remaining sections of this paper outline how this may be done in the US.

The rest of the paper is organized as follows: In Section II, we discuss the methods applied to clean and structure socioeconomic and COVID-19 data in a way to be applied to the models that are developed later on in the section. Following this, we validate the forecasting models and express key findings from our modeling approaches in Section III. Finally, we conclude the paper in Section V.

## II. Materials and Methods

### A. Forecasting Pipeline Overview

**Fig. 1.**
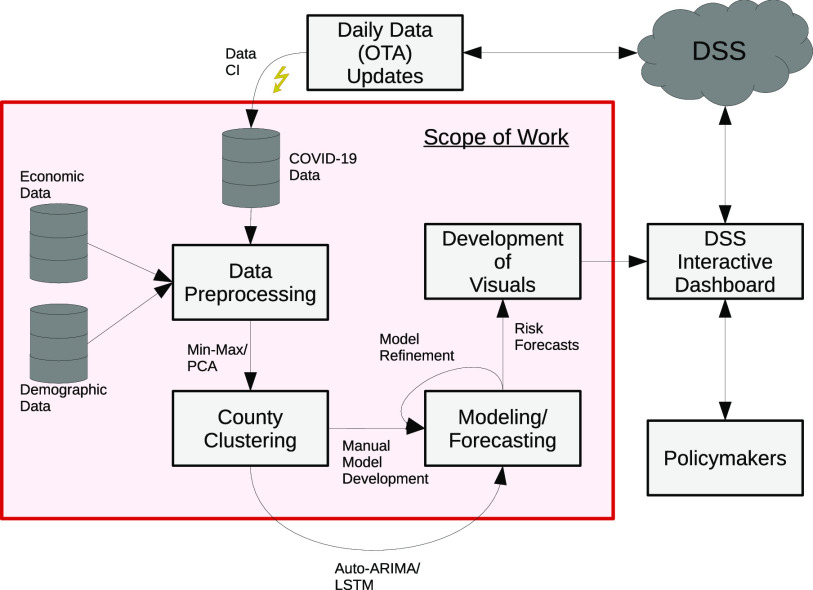
A comprehensive flowchart representing the proposed process of leveraging socioeconomic data for COVID-19 forecasting in a Decision Support System (DSS). The scope of the work in this paper is contained within the red square.

The incorporation of socioeconomic data with COVID-19 case and fatality data for a risk analysis Decision Support System (DSS) requires the development of a pipeline capable of preprocessing, refining, clustering, modeling, and visualizing the data. The processes required for the pipeline to work properly are visualized in Fig. 1. In this paper, we focus on the development of the core functions of the DSS – prepossessing the data, development of the clustering process to group the counties together based on socioeconomic similarity, manually developing time series forecasts for the data, and expressing the forecasts in visualizations that highlight the risk profiles for each cluster of counties.

### B. Data Preparation

#### 1) Dataset Overview

We have obtained several county-level datasets from the US. The first dataset contains demographic data such as population size, gender, racial, and age distributions for each county in the US. Note that for the scope of our analysis, we are only considering counties located within the 50 states, and excluding all non-state territories (i.e., Puerto Rico, US Virgin Islands, etc.) The demographic data are from the 2010 census and population trend forecasts for each year following the census, up to 2019, via the US Census Bureau. The second contains daily cumulative COVID-19 cases and deaths for each county in the United States from late March to late October 2020, roughly encompassing the first and second major infection waves, via the CDC. The third dataset contains United States county-, state-, and national-level Gross Domestic Product (GDP) from 2010 to 2018, via the US Bureau of Economic Analysis. The fourth dataset contains the GIS coordinates representing the geographic centers of each US county, along with their land areas in square miles, via the US Census Bureau. Finally, the fifth dataset is a lookup table that links each US state and county to a primary key for each county known as the county Federal Information Processing Standards (FIPS) codes that each of the aforementioned datasets are indexed row-wise by.

#### 2) Data Cleaning

As mentioned in the Introduction, we aim to cluster each US county-level territory based on similar demographic and economic profiles, as these counties are likely to contain groups of people with similar behaviors. In order to cluster these counties, we must perform an inner join on the five aforementioned datasets. Prior to performing the join, we must clean the data. Since we are looking at a public health crisis that occurs in 2020, we are only interested in the most recent data (2018/19). While we could apply time series models (like ARIMA or Holt-Winters Exponential Smoothing) to forecast the 2020 county-level demographics and Gross Domestic Product (GDP) contribution, in the interest of an expedient process due to the dire situation posed by the coronavirus, we are assuming that GDP in 2018 and 2019-level demographic data is a reasonable basis for clustering these counties together.

First, we aim to build the socioeconomic data table explained in Table I that will be used to cluster the counties together. We begin by isolating the most recent (2018) county-level GDP, and link the values to their corresponding FIPS key. We then generate two features from the raw GDP: the percentage of the state- and national-level GDP contributed by the county, by dividing the county GDP by the state and national GDP, respectively. We make one assumption when dealing with Virginia independent cities, because in the raw data, some were grouped together if they had small populations. If this was the case, we split them into two separate rows, and assumed they each contributed to half of the recorded GDP, as these were likely intertwined municipal economies. There are very few missing GDP data after these changes.

**TABLE I:** Refined Socioeconomic Dataset for County Clustering

**Feature**	**Description**
FIPS	FIPS (state + county)
sGDP	County percentage of state GDP
nGDP	County percentage of national GDP
rPOP	County Population (will not be used in clustering model)
sPOP	County population divided by state population
MALE	Percent of population that is male
FEMALE	Percent of population that is female
W_MALE	Percent of population that is White male
W_FEMALE	Percent of population that is White female
B_MALE	Percent of population that is Black male
B_FEMALE	Percent of population that is Black female
I_MALE	Percent of population that is Native American male
I_FEMALE	Percent of population that is Native American female
A_MALE	Percent of population that is Asian male
A_FEMALE	Percent of population that is Asian female
P_MALE	Percent of population that is Pacific Islander male
P_FEMALE	Percent of population that is Pacific Islander female
H_MALE	Percent of population that is Hispanic male
H_FEMALE	Percent of population that is Hispanic female
D_MALE	Percent of population that is 2+ race male
D_FEMALE	Percent of population that is 2+ race female
AAGE	Estimated average age, divided by 100
LAT	County geographic center latitude coordinate in degrees
LAT	County geographic center longitude coordinate in degrees
POP_DEN	County population density, in persons per square mile

After handling the economic data, we filter and clean the demographic data. We begin by selecting only the data from July 2019 – the most recent demographic forecasts. From the most recent demographic information, we select the columns containing age group sizes, and the populations of all men, all women, White men, White women, Black men, Black women, Native American men, Native American Women, Hispanic/Latino men, Hispanic/Latino women, men identifying as two or more races, and women identifying as two or more races for each age group, respectively. These features are linked to their respective county FIPS code. The age groups are the number of people binned in half-decade increments (i.e., 0–4, 5–9, 10–14,..., 85+ years old). From the selected features, we compute the percentage of the county belonging to an ethnic/racial group by taking each of the aforementioned demographic groups and dividing them by their county's respective total population count. The average age of the county is estimated by taking a weighted average of the sizes of the stratified age groups of the county data. The average age is normalized by dividing by 100. Finally, in order to attain the county population density in persons per square mile, we divide the estimated county population by the land area of the county in square miles. Following the economic and demographic preprocessing, we then combine the features into the demographic table by performing an inner join along the FIPS key. This results in the refined structured data summarized in Table I.

We then turn our attention to the county-level COVID-19 data. We isolate only the features containing the FIPS key, date of data recording, the number of cumulative cases, and the number of cumulative fatalities based on the FIPS key. We then divide the number of cumulative cases/fatalities for each day in the county time series by the respective county population. This leads to the time series to be represented as a percentage rather than an absolute number of cases, allowing for a per-capita analysis of the COVID-19 pandemic progression that leads to ease of comparison. This processed time series data is summarized in Table II.

**TABLE II:** Refined COVID-19 County-Level Infection and Fatality Dataset for Infection Modeling

**Factor**	**Description**
FIPS	FIPS (state + county)
DATE	Date of case/death measurement
pINFECTED	Cumulative county population percentage infected
pDEAD	Cumulative county fatality percentage

After generating the clean data organized by the structure defined in Tables I and II, we perform two last preprocessing steps on the demographic data. First, we perform min-max normalization on each feature in the demographic data in order to ensure that all of the features exist along the same $[0, 1]$ scale. Following this, we then perform Principal Component Analysis (PCA) [33] on the normalized demographic/economic data. Due to the fact that much of the demographic information is likely to contain correlated information, we apply PCA (via the python sklearn package [34]) to reduce the dimensionality of the data and retain most (in this work, 99.5%) of the variance of the data. This will aid in facilitating the development of clusters that more readily capture distinctions. PCA also helps to counter the curse of dimensionality. As the number of features increases, the feature space becomes sparser, reducing the effectiveness of Euclidean distance as a measure for the clustering approaches. These refined data will be utilized in the development of the two cornerstone models in this prediction system.

### C. Model Development

In this section, we develop the models that are utilized in the demographic-based forecasting approach. First, we utilize the preprocessed demographic data to form clusters of counties with the aim that they exhibit maximized demographic similarity. Following the grouping of counties together, we then pool their cumulative infection and fatality data to create unique infection and fatality time series for each of the aggregated groups. Following this, we develop ARIMA forecasts for each of the aggregated clusters, which are then utilized to gauge future COVID-19 risk.

#### 1) County Clustering Approaches

The US is vast and diverse, both geographically and demographically. Due to this, when modeling a pandemic in the US, it is worthwhile to break the country up into regions, and model the spread in each region. Oftentimes, we consider geographical regions when subdividing the US (i.e., NJ, NY, CT, and PA are often grouped together as the Mid-Atlantic States, due to their geographic proximity to the Atlantic Coast of the United States, and position between New England and the Southern United States). Due to the rapid nature in which Americans can easily traverse the United States (e.g., one can fly from New York City to West Palm Beach, a span of nearly 1500 miles, in less than three hours), and the ability for the virus to be transmitted via asymptomatic carriers, we think that there are other factors that link the transmission of the virus more effectively than geography. Economic and demographic factors, in particular, may offer a pathway to developing a more robust system to group American counties together. For example, Los Angeles County, CA is likely to be more similar in the context of this pandemic to New York County (Manhattan), NY than Imperial County, CA. While Imperial County is located much closer to LA county than New York County, LA county likely has a much more similar economic and demographic profile with Manhattan than the agricultural Imperial County. These economic and demographic similarities more likely than not imply similar population movement and dispersion patterns than simple geography. In addition, the effects of the virus may be more likely to lead to different prognoses based on race. Indeed, studies have shown that Black and Hispanic Americans are hospitalized [35] and die from COVID-19 [36] at higher rates than White Americans.

To this end, we apply clustering approaches to the PCA-feature engineered dataset described in Table I, in order to develop “regions” that are linked via racial/ethnic composition and economic productivity (which correlates with population mobility, size, and wealth), in addition to geographic considerations. In clustering approaches, the aim is to determine $k$ centroid points in the data such that the sum of the variance of the distance between points in a cluster and its centroid (i.e., Sum of Squared Errors (SSE)) are minimized – this can be mathematically expressed as follows:

\begin{equation*}
SSE = \min \sum _{c = 1}^{k}\sum _{i \in \mathcal {C}_{k}} ||x_{i} - \mu _{c}||^{2}, \tag{1}
\end{equation*}

where $x_{i}$ is a data entry, $\mu _{c}$ is the centroid of cluster $c$, $\mathcal {C}_{c}$ is the set of entries that are contained within cluster $c$ such that each county $i$ is only contained within one cluster.

There exist several algorithms to find suboptimal solutions to the NP-hard problem expressed in (1). The most widely-known and used unsupervised approach is the naive $k$-means algorithm [37]. Other approaches leverage metaheuristic search algorithms such as Particle Swarm Optimization (PSO) to search for the cluster centroids [38].

In order to determine a reasonably good set of clusters in the context of this problem, we utilize the elbow and silhouette analysis methods to evaluate the effectiveness of the clusters. For both, we first run the PSO and $k$-means approaches for a varying number of $k$ clusters ($2-20$). In the elbow method, we compute the sum of squared errors for each set of clusters as expressed in (1), and subjectively look to see where a diminishing returns effect in the performance gains occur (the “elbow”) in the graph. For the silhouette analysis method [39], we evaluate the silhouette coefficients for each number of clusters ($2-20$), where the coefficient is a value in the $[-1, 1]$ range. A value of 1 for a silhouette means that the point fits the cluster very well and is located far away from the boundary for another cluster, a value close to 0 implies that the point lies close to the boundary of another cluster, and a negative value implies that a point may be located in the wrong cluster.

**Fig. 2.**
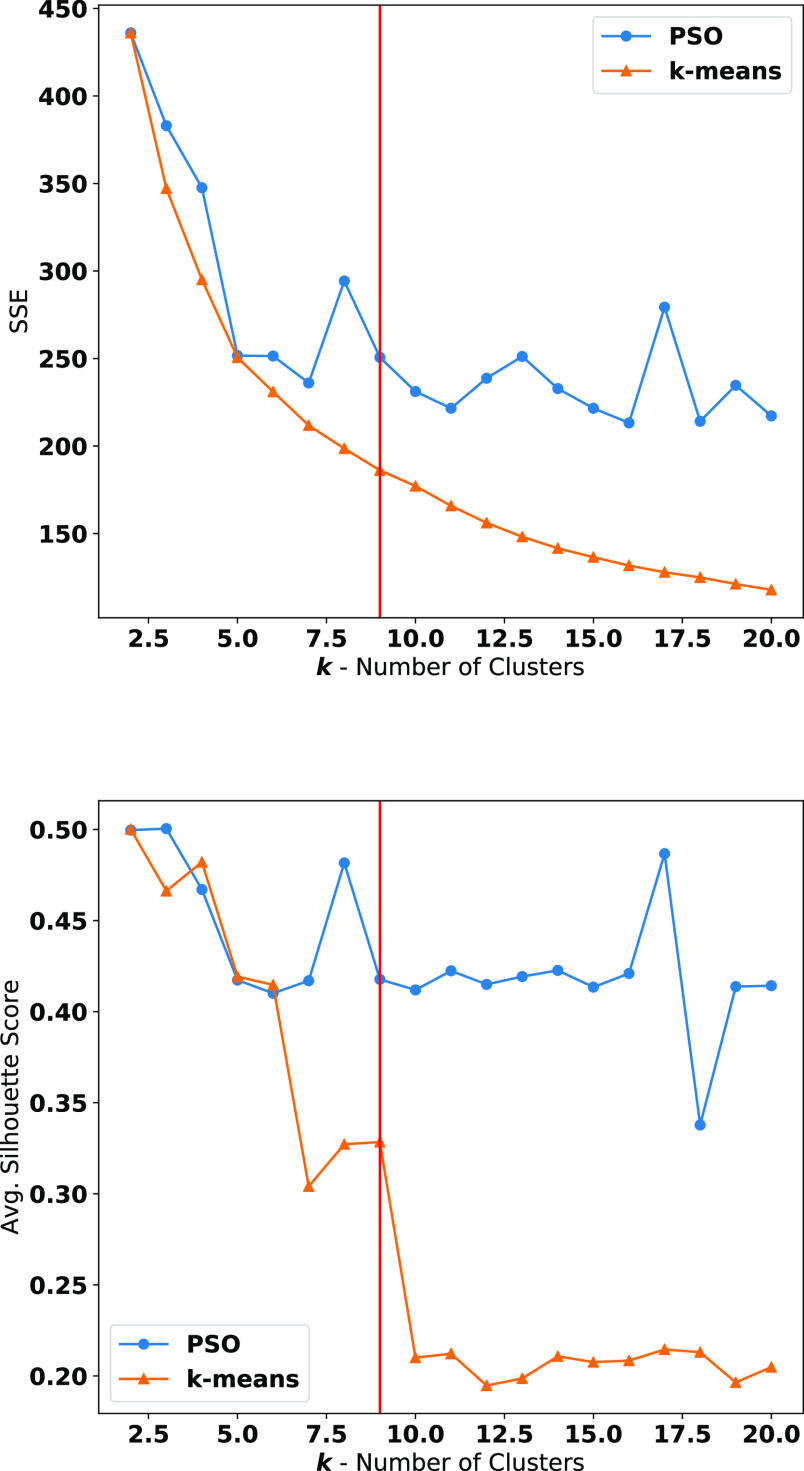
(Top) Sum of Squared Errors (SSE) for each number of clusters $k$, calculated from (1). (Bottom) Average silhouette coefficient for each number of clusters $k$. The red vertical lines correspond to the selected number of clusters ($k=9$).

**Fig. 3.**
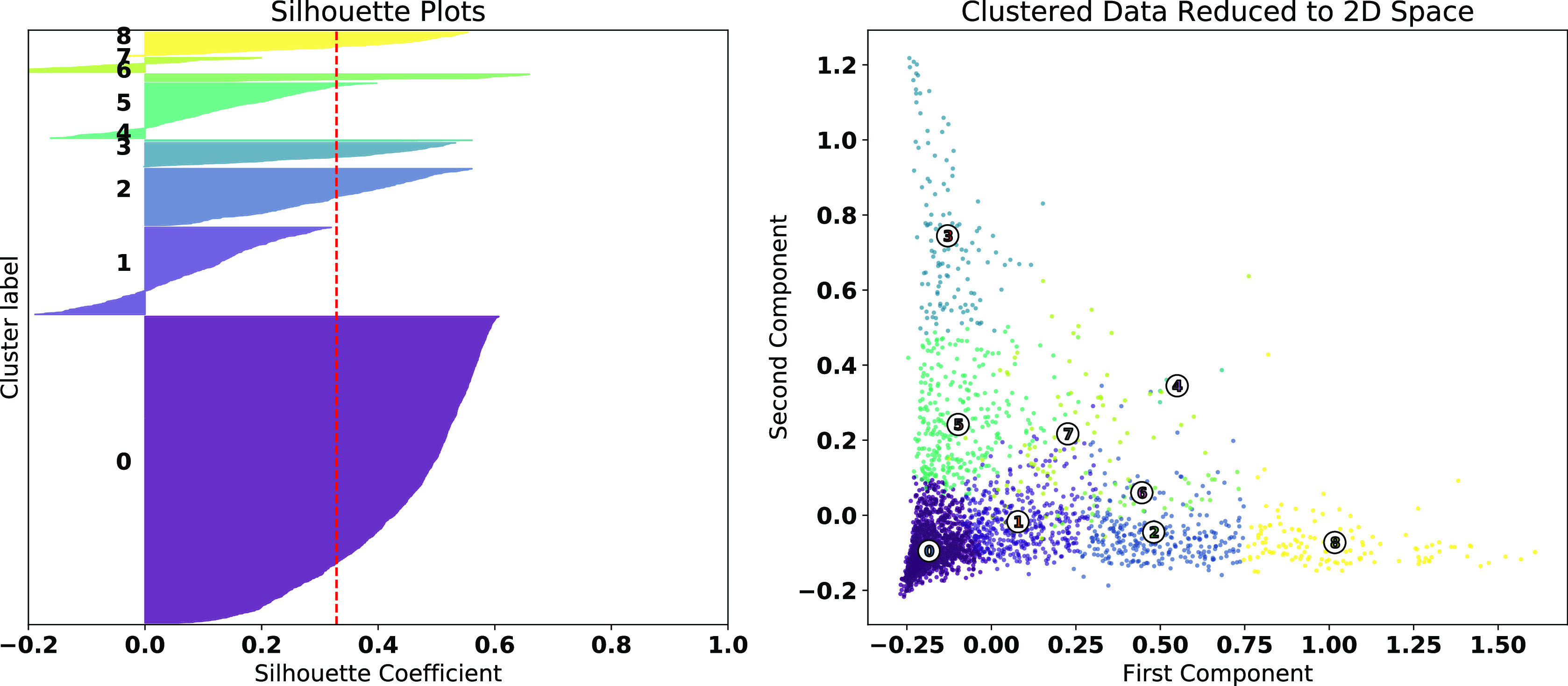
(Left) Silhouette plots for $k=9$ clusters. The dashed red vertical line corresponds with the average silhouette score. (Right) Clustering dataset compressed to two-dimensional space with PCA to visualize the effectiveness of the clusters. The clusters are color-coded and their centroid is numerically labeled. We notice that some of the lower silhouette scores result from the decision boundaries between a handful of clusters, however clearly-defined regions appear to have been formed.

Based on the results of the elbow analysis and the average silhouette scores for each $k$ presented in Fig. 2, we selected $k=9$ as the most appropriate, as we felt that it appropriately handled the trade-off between low SSE, acceptable average silhouette coefficient value, and had a sufficiently large enough number of distinct clusters to draw nuance in the county cluster demographics. While the average silhouette coefficient of approx. 0.35 is somewhat low, in the context of the data we have it appears to generate sufficiently good clusters. We see in Fig. 3 that while Clusters 1, 5, and 7 have some members with negative silhouette values, the clusters formed appear to have relatively well-defined regions.

**Fig. 4.**
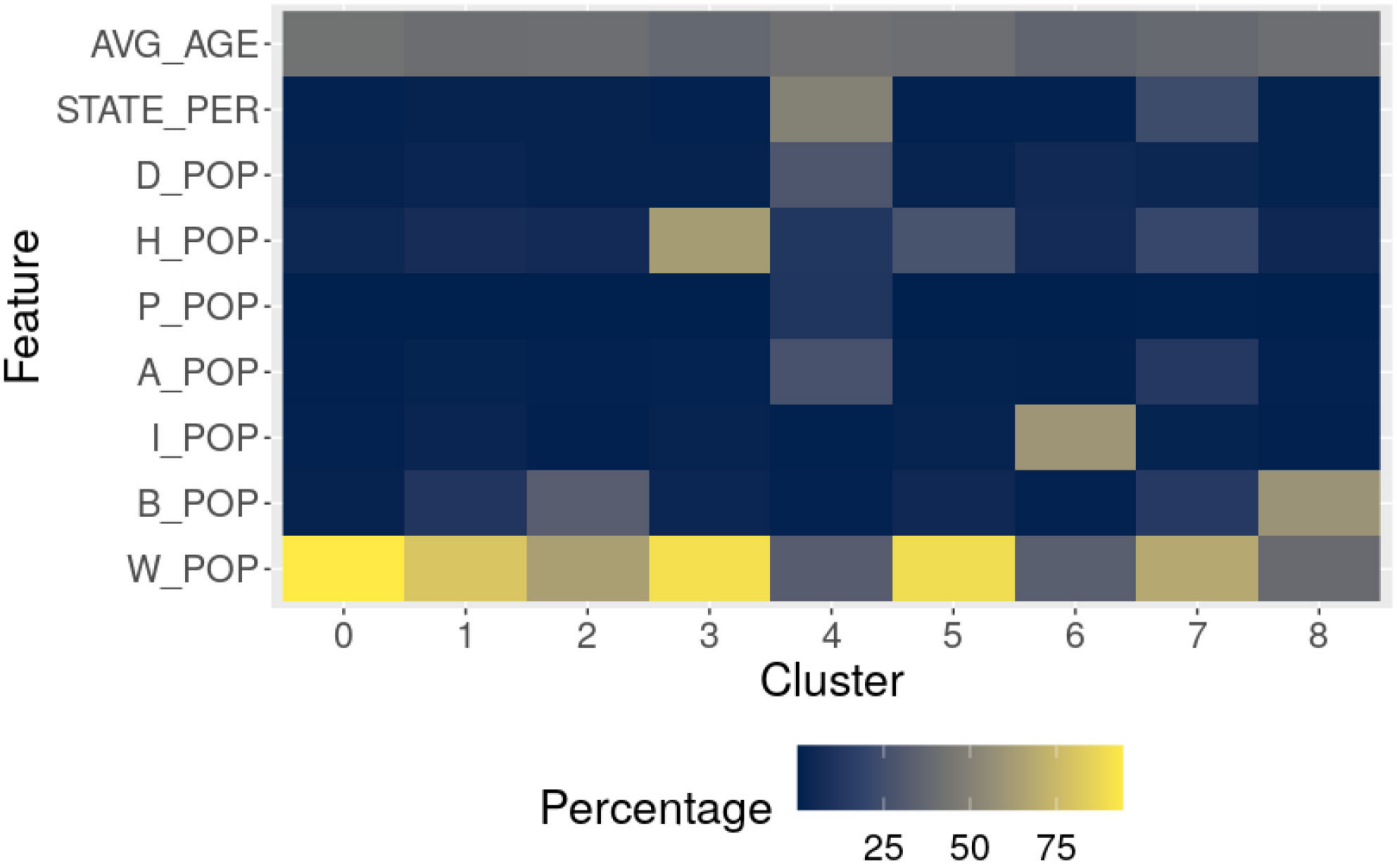
Heatmap of the county centroid values. Darker values signify lower percentages. These values are utilized in conjunction with Table III to express the profiles of the county clusters.

**Fig. 5.**
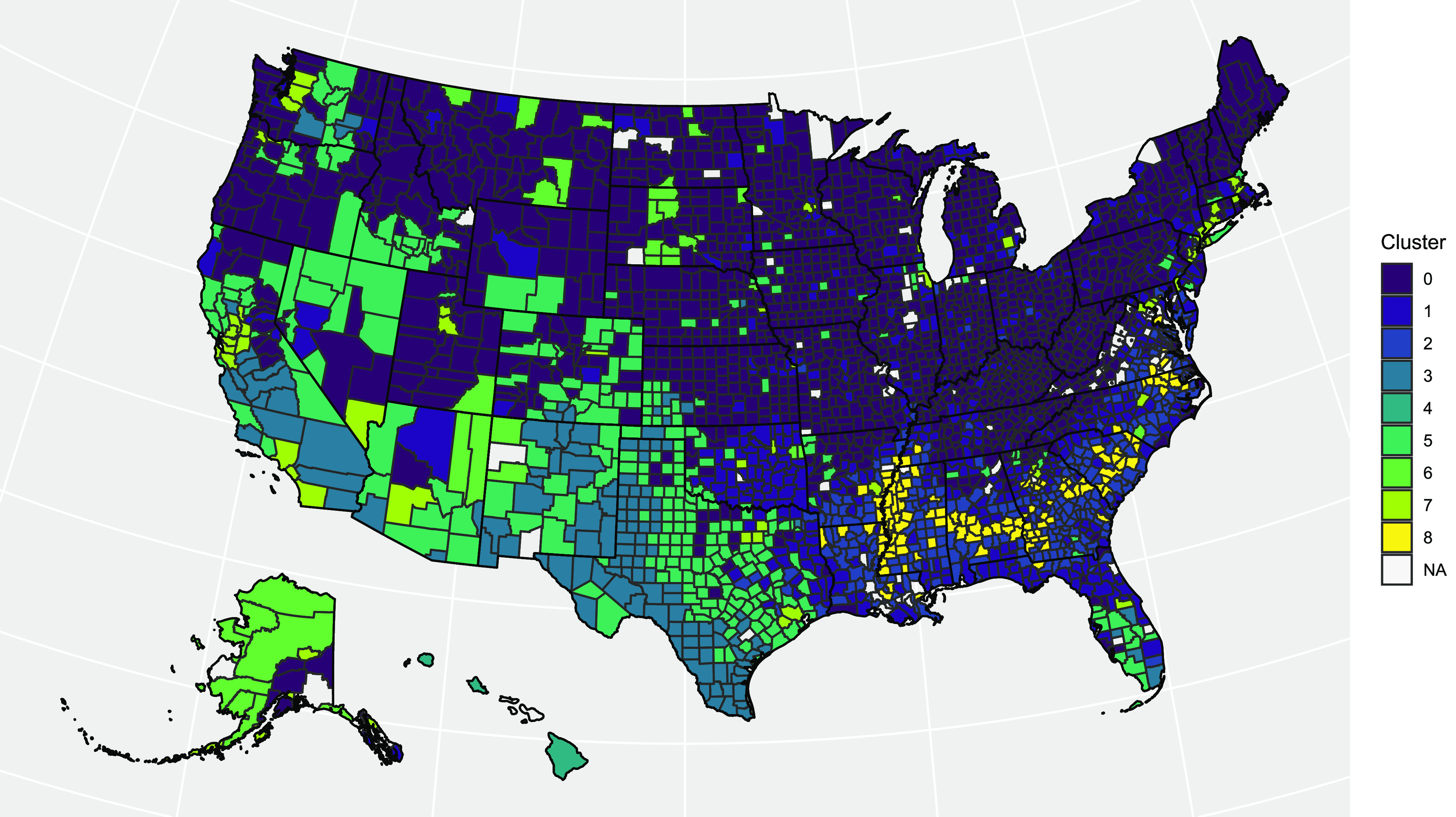
Visual representation of county groups, where the color corresponds to the cluster determined by $k$-Means Clustering. Blank counties imply that we do not have data for them.

We summarize the profiles of each county cluster briefly in Table III, provide example counties that are most like the cluster centroid in Table III, and provide the cluster geographic centers, populations, and population densities in Table III. The cluster profiles are constructed based on the mean values for each cluster centroid. Fig. 4 shows that each cluster has a distinct demographic profile. Interestingly, while we did not account for geography at all in our clustering process, the clusters show some geographical connectedness, and unique regional positioning (i.e., a majority of counties with a majority Black population are located within the deep south states Louisiana, Arkansas, Mississippi, Alabama, Georgia, and South Carolina). implying that demographics and economic productivity correlate with location in the US. This is visualized with a map that color codes each county based on its assigned cluster in Fig. 5.

**TABLE III:** County Cluster Profiles Based on Fig. 4

**Cluster**	**Description**
0	White-majority counties
1	White-majority counties with a small Black plurality
2	White-majority counties with a significant Black plurality
3	Latino-majority counties
4	Counties located within the state of Hawaii
5	White-majority counties with a significant Latino plurality
6	Native American-majority counties
7	Diverse, major metropolitan areas
8	Black-majority counties

#### 2) Time Series Modeling of Cases and Deaths

After the clustering process in which we combined the cumulative COVID-19 case and death data for each cluster, we apply time series analysis methods in order to build forecasts of future trends in the data. These forecasted trends are the basis for the risk modeling, as areas that are expected to have dramatic increases in cases, and consequently, deaths, are likely to need more resources to manage their outbreaks. Prior to the development of the models, we first develop the infection and fatality time series for each of the clusters generated previously. We generate the series by computing the weighted sum of the percentages of the counties in each cluster infected/dead, and divide the sums by the total population of the cluster of counties to obtain the cumulative percentage infected or dead by COVID-19 in the cluster. After obtaining the cumulative fraction of infections/fatalities to the cluster population, we then take the first-order difference (i.e., the cumulative cases from one day minus the previous day) to obtain the daily new active infections/fatalities. The new active infections/fatalities serve as the series of interest to model.

Seasonal Autoregressive Integrated Moving Average with eXogenous factors (SARIMAX) models are generalized versions of the Autoregressive Integrated Moving Average (ARIMA) model [40]. ARIMA is a powerful, explainable modeling approach for forecasting time series. SARIMAX improves upon ARIMA by incorporating seasonality and external influences on a time series. The consideration for seasonality is a result of how COVID-19 data collection and reporting occurs in practice – there is weekly seasonality in both the cumulative cases and fatalities series as more data is collected/processed during the work week versus the weekend. When modeling the cumulative cases series, we consider the series as independent endogenous factors, and utilize a SARIMA (no exogenous factors) approach. It is worthwhile to note that fatalities are a lagging indicator from the number of cases, as it typically takes 2-3 weeks for increases in deaths to result from increases in cases, based on symptomatic progression of the disease [41]. We thus utilize SARIMAX approaches for fatalities, with cumulative cases as an exogenous factor.

In order to streamline the discussion of the SARIMA/SARIMAX models, we will refer to them collectively as ARIMA models, and utilize the following notation to express the model orders: ARIMA$(p, d, q)x(P, D, Q, s)$, where $p$ represents the autoregressive order of the model, $d$ represents the number of first-order differences applied to the series, $q$ represents the moving average order of the model, and finally, $P$, $D$, and $Q$ refer to the seasonal autoregressive, difference, and moving average orders for a seasonality of $s$ lags, respectively. In the context of this problem, we utilize a weekly seasonality ($s = 7$).

*a) ARIMA Forecasting*. After constructing the infection and fatality time series for each of the clusters, we explore and fit their corresponding individual ARIMA models. We note that after performing the two first-order differences and one first-order seasonal difference on the series in order to achieve weak stationary series, there still existed autocorrelations in the de-trended data residuals. In order to address this, we fit separate ARIMA models for each time series based on manual analysis of the residual patterns. From the analysis of the time series for each cluster, we have fit ARIMA$(0, 1, 1)x(0, 1, 1, s=7)$ models to the active case series for each cluster. We also fit ARIMA$(1, 0, 0)x(0, 1, 1, s=7)$ models to the fatalities series for each cluster, with infections from 14 days prior as exogenous factors. In other words, we include a linear relationship between the daily change in fatalities and the daily change in cases 14 days prior along with the ARIMA components.

*b) Seasonal Trend Random Walk Forecasting*. A random walk process is a discrete stochastic process, where at each interval, the state of the process has an independent likelihood of increasing or decreasing [42]. In the context of time series models, this implies that once de-trended, there exists no autoregressive or moving average components; each observation is independent of the previous one. In the context of ARIMA, a random walk may be modeled as ARIMA$(0, 1, 0)$, and if there is a seasonal trend as well, then it may be modeled as (ARIMA$(0, 1, 0)x(0, 1, 0, s)$) [43]. When analyzing the trends of the COVID-19 case and death time series, it is notable that in order to de-trend the series (i.e., make the series approximately stationary), a first-order difference and a first-order seasonal difference can be applied to the active case and death series, where the seasonality is weekly due to how COVID-19 data is collected and reported.

*c) Forecasting Approach*. Pandemics lead to very rapidly changing circumstances. Due to the exponential growth in the number of infections that may occur in uncontrolled settings, the forecasts for the number of new active cases and fatalities must regularly be updated. In our analysis, we simulate this re-calibration on the data by utilizing a sliding window of interest. Our models will output 28-days forecasts from 56 days, worth of training data. We then slide the window forward every two weeks. In a real-life scenario, this would be equivalent to producing the first forecast after 8 weeks of recorded transmissions, and re-calibrating the model every two weeks. We test the forecast accuracy by comparing the first seven days of the forecast with the true data in each forecast window. This sliding window approach is utilized to measure the effectiveness of the models introduced earlier in this section.

### D. Validation Metrics

An effective method for validating the fit of ARIMA models is a Ljung-Box goodness-of-fit test on the model residuals [44]. In this test, the null hypothesis is that the residuals are not autocorrelated, and the alternative is that they are. Autocorrelations in the residuals imply that the model has not fully captured the signal in the data or that the model is over-fitting to noise. A general rule of thumb is to apply the test to the lag-$\text{2}~s$ residual in ARIMA models, where $s$ is the seasonality. In the context of our model, we apply this to the 14$^\text{th}$ lag. The results of the test are summarized in Table VI. In order to verify that the re-calibration forecasting strategy outlined in Paragraph II-C2 leads to models that fit the data well, we need to track whether or not the sub-models fail to reject the null hypothesis at the $\alpha = 0.01$ significance level. To this end, we check to see the total number of times where the results of the Box-Ljung test led to this outcome out of the 16 total sliding window forecasts. Based on the results presented in Table VI, the ARIMA models provide much stronger fits than the random walks. In every cluster, several more of the sliding window sub-models for the ARIMA forecasts failed to reject the null hypothesis versus the random walk models. This implies that the ARIMA models are much more effective at capturing the signal in the time series than the Random Walk models.

**TABLE IV:** Counties That are Closest to the Mean of the Cluster Distribution

**Cluster**	**Profile County**
0	Lee County, Iowa
1	Limestone County, Alabama
2	Union County, Arkansas
3	Yuma County, Arizona
4	Hawaii County, Hawaii
5	Wheeler County, Texas
6	Roosevelt County, Montana
7	Montgomery County, Maryland
8	Jefferson County, Arkansas

**TABLE V:** Other Cluster-Related Information – Geographic Centers and Average Population Densities for Each Cluster

	**Geographic Center**	**Density**	**Population**
0	40.9328$^{\circ}$N, 92.0760$^{\circ}$W	77.40/mi$^2$	79 393 206
1	36.3866$^{\circ}$N, 87.7773$^{\circ}$W	313.16/mi$^2$	62 076 955
2	33.7166$^{\circ}$N, 84.8526$^{\circ}$W	332.02/mi$^2$	32 638 877
3	32.7301$^{\circ}$N, 103.5772$^{\circ}$W	83.58/mi$^2$	20 949 435
4	20.3107$^{\circ}$N, 156.6530$^{\circ}$W	323.22/mi$^2$	1 248 369
5	35.8153$^{\circ}$N, 101.4713$^{\circ}$W	156.07/mi$^2$	30 508 636
6	47.5705$^{\circ}$N, 114.2894$^{\circ}$W	10.75/mi$^2$	752 702
7	40.3335$^{\circ}$N, 93.5081$^{\circ}$W	6718.92/mi$^2$	81 859 375
8	33.6025$^{\circ}$N, 85.1336$^{\circ}$W	417.57/mi$^2$	10 471 296

**TABLE VI:** Ljung-Box Test Results At lag-14. We Determine the Number of Rolling Horizon Forecasts Where We Fail to Reject the Null Hypothesis of Random Autocorrelation (Out of Sixteen Total) At the $\alpha =0.01$ Level. The Better Performing Model is Highlighted in **Boldface** Font

**Cluster**	**ARIMA**		**Random Walk**	
	**Cases**	**Deaths**	**Cases**	**Deaths**
0	$\mathbf {13}$	12	6	12
1	$\mathbf {16}$	$\mathbf {14}$	5	8
2	$\mathbf {13}$	$\mathbf {15}$	7	10
3	$\mathbf {8}$	$\mathbf {14}$	3	11
4	$\mathbf {13}$	$\mathbf {12}$	3	5
5	$\mathbf {14}$	$\mathbf {14}$	4	10
6	$\mathbf {12}$	$\mathbf {12}$	0	9
7	$\mathbf {14}$	$\mathbf {13}$	4	11
8	$\mathbf {13}$	$\mathbf {14}$	3	8

Another useful metric for evaluating the goodness-of-fit of the model is the Root Mean Squared Error (RMSE), which can be determined as follows:

\begin{equation*}
{RMSE_{cm} = \sqrt{\frac{1}{(T - t^{*}_{cm})}\sum _{t = t^{*}_{cm}}^{T}{(y_{cm}^{t} - \hat{y}_{cm}^{t})^2}},} \tag{2}
\end{equation*}

where $RMSE_{cm}$ is the Root Mean Squared Error for the model type $m=\lbrace \text{cases}, \text{deaths}\rbrace$ fitted for cluster $c$, $t^{*}_{cm}$ is the index of the first day of recorded infections or deaths, respectively, for the series that is fitted for cluster $c$, $T$ is the index of the day that splits the forecast from the fit, $\hat{y}_{cm}^{t}$ are the fitted values in the time series model, and $y_{cm}^{t}$ is the real data. Note that $y_{cm}^{t} - \hat{y}_{cm}^{t}$ are the residual values. The RMSE scores for the daily new active infections and fatalities are summarized in Tables VII and VIII, respectively.

**TABLE VII:** RMSE of the Active Infection Model Fits. The Model With the Better (lower) RMSE is Highlighted in **Boldface** Font

	**ARIMA**			**Random Walk**		
	**Best**	**Worst**	**Avg.**	**Best**	**Worst**	**Avg.**
0	0.0064	$\mathbf {1.5065}$	$\mathbf {0.6992}$	$\mathbf {0.0063}$	1.8044	0.8246
1	$\mathbf {0.0032}$	$\mathbf {2.1879}$	$\mathbf {1.0248}$	0.0033	2.7002	1.2055
2	0.0561	4.3354	$\mathbf {1.8296}$	$\mathbf {0.0043}$	$\mathbf {4.2330}$	2.1262
3	$\mathbf {0.0074}$	$\mathbf {9.0174}$	$\mathbf {3.5641}$	0.0102	11.9887	4.4232
4	$\mathbf {0.0160}$	$\mathbf {4.6405}$	$\mathbf {1.5147}$	0.0227	6.2542	2.0357
5	$\mathbf {0.0044}$	$\mathbf {2.9382}$	$\mathbf {1.8167}$	0.0045	4.1838	2.3303
6	0.0000	$\mathbf {12.7034}$	$\mathbf {6.6349}$	0.0000	17.5091	9.5875
7	0.0223	$\mathbf {3.5187}$	$\mathbf {1.6922}$	$\mathbf {0.0203}$	5.4394	2.2002
8	0.0169	$\mathbf {4.8051}$	$\mathbf {3.0213}$	$\mathbf {0.0122}$	6.2978	3.7096

**TABLE VIII:** RMSE of the Active Fatality Model Fits. The Model With the Better (lower) RMSE is Highlighted in **Boldface** Font

	**ARIMA**			**Random Walk**		
	**Best**	**Worst**	**Avg.**	**Best**	**Worst**	**Avg.**
0	$\mathbf {0.0002}$	$\mathbf {0.0948}$	$\mathbf {0.0556}$	0.0003	0.0968	0.0592
1	$\mathbf {0.0005}$	$\mathbf {0.1957}$	$\mathbf {0.0988}$	0.0006	0.2183	0.1171
2	0.0000	$\mathbf {0.2023}$	$\mathbf {0.1313}$	0.0000	0.3087	0.1658
3	0.0000	$\mathbf {0.4718}$	$\mathbf {0.1497}$	0.0000	0.6089	0.1946
4	0.0000	$\mathbf {0.2414}$	$\mathbf {0.0689}$	0.0000	0.3429	0.0989
5	$\mathbf {0.0008}$	$\mathbf {0.2331}$	$\mathbf {0.1403}$	0.0011	0.2824	0.1644
6	0.0000	$\mathbf {1.0899}$	$\mathbf {0.5199}$	0.0000	1.2757	0.6925
7	$\mathbf {0.0013}$	$\mathbf {0.2796}$	$\mathbf {0.1560}$	0.0015	0.4160	0.1768
8	0.0000	$\mathbf {0.3435}$	$\mathbf {0.1798}$	0.0000	0.4367	0.2105

In addition to evaluating the goodness of fit of a model, it is worthwhile to utilize metrics to evaluate the effectiveness of a forecast. In the context of time series models, measures of the effectiveness of a forecast should measure the bias and the accuracy of the model. An effective metric for measuring the forecast accuracy is the Mean Absolute Error (MAE), expressed as follows:

\begin{equation*}
{MAE_{cm} = \frac{1}{(T_{f} - T)}\sum _{t = T}^{T_{f}}{\left|y_{cm}^{t} - \tilde{y}_{cm}^{t}\right|},} \tag{3}
\end{equation*}

where $MAE_{cm}$ is the MAE for each model forecast, $\tilde{y}_{cm}^{t}$ are the forecasted values, and $T_{f}$ is the index of the $f$-step ahead forecast. In the context of the models proposed in this paper, we validate on the one week, or $f=7$ forecast. A smaller MAE implies a more accurate model. The MAE scores for the daily new active infections and fatalities are summarized in Tables IX and X, respectively.

**TABLE IX:** MAE of the Active Infection Model Fits. The Model With the Better (lower) MAE is Highlighted in **Boldface** Font

	**ARIMA**			**Random Walk**		
	**Best**	**Worst**	**Avg.**	**Best**	**Worst**	**Avg.**
0	0.0355	$\mathbf {3.2862}$	1.1835	$\mathbf {0.0269}$	3.4109	$\mathbf {1.1604}$
1	$\mathbf {0.0720}$	$\mathbf {3.5562}$	$\mathbf {1.5238}$	0.0738	3.3965	1.5465
2	0.7774	4.8982	2.1578	$\mathbf {0.1228}$	$\mathbf {4.1804}$	$\mathbf {2.1275}$
3	0.0634	$\mathbf {16.7885}$	$\mathbf {3.4999}$	$\mathbf {0.0368}$	30.3101	4.8000
4	$\mathbf {0.0955}$	$\mathbf {4.5484}$	$\mathbf {1.5462}$	0.1144	14.7736	2.4408
5	0.0847	4.7425	$\mathbf {2.3754}$	$\mathbf {0.0735}$	$\mathbf {4.7189}$	2.5162
6	0.0190	$\mathbf {10.3001}$	$\mathbf {6.3745}$	0.0190	24.6878	9.6583
7	$\mathbf {0.1225}$	4.6037	$\mathbf {1.9850}$	0.1562	$\mathbf {4.0214}$	2.3663
8	0.3150	$\mathbf {7.1010}$	$\mathbf {3.3735}$	$\mathbf {0.2033}$	7.3449	3.7812

**TABLE X:** MAE of the Active Fatality Model Fits. The Model With the Better (lower) MAE is Highlighted in **Boldface** Font

	**ARIMA**			**Random Walk**		
	**Best**	**Worst**	**Avg.**	**Best**	**Worst**	**Avg.**
0	$\mathbf {0.0007}$	0.1568	0.0676	0.0008	$\mathbf {0.1430}$	$\mathbf {0.0655}$
1	$\mathbf {0.0018}$	$\mathbf {0.2201}$	$\mathbf {0.1035}$	0.0036	0.2486	0.1097
2	0.0013	0.4124	0.1564	0.0013	$\mathbf {0.3456}$	$\mathbf {0.1559}$
3	0.0027	0.5836	$\mathbf {0.1427}$	0.0027	$\mathbf {0.5781}$	0.1838
4	0.0000	0.4450	$\mathbf {0.0875}$	0.0000	$\mathbf {0.3411}$	0.0912
5	0.0028	$\mathbf {0.3647}$	$\mathbf {0.1352}$	0.0028	0.6638	0.1757
6	0.0000	$\mathbf {1.0564}$	$\mathbf {0.4484}$	0.0000	1.6289	0.6413
7	0.0037	0.5020	$\mathbf {0.1402}$	0.0037	$\mathbf {0.4008}$	0.1591
8	0.0068	0.5915	$\mathbf {0.2083}$	0.0068	$\mathbf {0.4442}$	0.2182

To measure the bias of the forecast, a useful metric is the Mean Error (ME), expressed as follows:

\begin{equation*}
{ME_{cm} = \frac{1}{(T_{f} - T)}\sum _{t = T}^{T_{f}}{(y_{cm}^{t} - \tilde{y}_{cm}^{t})},} \tag{4}
\end{equation*}

where $ME_{cm}$ is the ME for each model forecast. A negative bias implies that the forecast is overestimating the true trend, and a positive bias implies the forecast is underestimating the true trend. The ME scores for the daily new active infections and fatalities are summarized in Tables XI and XII, respectively.

**TABLE XI:** ME of the Active Infection Model Fits. The Model With the Better (Closest to Zero) ME is Highlighted in **Boldface** Font

	**ARIMA**			**Random Walk**		
	**Best**	**Worst**	**Avg.**	**Best**	**Worst**	**Avg.**
0	0.022	2.192	$\mathbf {-0.017}$	$\mathbf {0.016}$	$\mathbf {-1.396}$	$-0.315$
1	0.069	$-2.507$	$\mathbf {-0.364}$	$\mathbf {-0.027}$	$\mathbf {-2.437}$	$-0.693$
2	$\mathbf {-0.023}$	$-4.898$	$\mathbf {-0.325}$	$-0.120$	$\mathbf {-3.294}$	$-0.511$
3	0.063	$\mathbf {-16.788}$	$\mathbf {-1.047}$	$\mathbf {0.029}$	$-30.310$	$-1.370$
4	0.046	$\mathbf {2.929}$	$\mathbf {0.205}$	$\mathbf {-0.004}$	14.774	0.581
5	$\mathbf {-0.031}$	$\mathbf {-3.977}$	$\mathbf {-0.441}$	0.073	$-4.288$	$-0.699$
6	0.019	$\mathbf {-7.830}$	$\mathbf {-0.622}$	0.019	24.688	2.600
7	$\mathbf {-0.021}$	$-4.604$	$\mathbf {-0.503}$	0.155	$\mathbf {4.021}$	$-0.990$
8	$\mathbf {0.134}$	$-7.101$	$\mathbf {-0.902}$	0.184	$\mathbf {-6.320}$	$-1.512$

**TABLE XII:** ME of the Active Fatality Model Fits. The Model With the Better (Closest to Zero) ME is Highlighted in **Boldface** Font

	**ARIMA**			**Random Walk**		
	**Best**	**Worst**	**Avg.**	**Best**	**Worst**	**Avg.**
0	$\mathbf {0.000}$	$-0.157$	$-0.021$	$-0.001$	$\mathbf {0.143}$	$\mathbf {-0.008}$
1	0.000	$-0.220$	$-0.029$	0.000	$\mathbf {0.208}$	$\mathbf {0.012}$
2	$\mathbf {0.000}$	$-0.412$	$-0.045$	0.001	$\mathbf {-0.230}$	$\mathbf {0.012}$
3	0.003	$\mathbf {0.118}$	$\mathbf {-0.001}$	0.003	$-0.457$	$-0.023$
4	0.000	$\mathbf {0.251}$	$\mathbf {-0.016}$	0.000	$-0.341$	$-0.033$
5	0.002	$\mathbf {-0.261}$	$\mathbf {-0.043}$	0.002	$-0.620$	$-0.062$
6	0.000	$\mathbf {-0.472}$	$\mathbf {-0.054}$	0.000	1.629	0.056
7	0.001	$-0.502$	$-0.067$	0.001	$\mathbf {-0.386}$	$\mathbf {-0.033}$
8	0.007	$-0.529$	$-0.072$	$\mathbf {0.001}$	$\mathbf {-0.444}$	$\mathbf {-0.024}$

Note that when computing the validation metrics, the values of the time series expressed as infections/deaths per 100 k are used. In other words, an MAE score of 1 implies an average error magnitude of 1 infection per 100 k in the forecast window.

## III. Results

In this section, we validate the approach of the random walk and ARIMA forecasts developed in the previous section. We implement the fitting process for the Seasonal Trend Random Walk, and custom ARIMA$(p, d, q)x(P, D, Q, s)$ for each cluster of counties (via the python numpy, pandas, and statsmodels packages [45]–[48]).

Based on the results of our residual analysis via the Box-Ljung test, along with the RMSE, MAE, and ME metrics, the ARIMA models for each cluster are superior to the random walk models. For each cluster, there are more sub-forecasts in the simulated biweekly re-calibration where the ARIMA models fail to reject the null hypothesis of the Box-Ljung test at the $\alpha = 0.01$ significance level versus the random walk models. This implies that over time, the ARIMA models are more effective at capturing the signal of the time series processes in each cluster versus the random walk models. The results of the RMSE calculations further reinforce this conclusion – the average RMSE for each cluster's ARIMA models are lower than those of the random walk, showing that the fitted time series curve more closely approximates the true data. In most clusters, the average MAE and ME of the ARIMA models were lower/closer to zero, respectively, implying more accurate and less biased forecasts than the random walk counterparts. Based on the results of the residual analysis, we utilize the ARIMA models for the remainder of the analysis conducted in the manuscript.

### A. Cluster Risk Modeling

After the validation and model-selection process, we apply the fitted ARIMA models to each of the clusters and generate their 28-day forecasts such that the first seven days of the forecast overlap with the last seven days of real data and the remaining 21 days of the forecast extends beyond the most recent data. This is done to show visually whether or not the fitted models adequately forecast future trends, and to provide a visualization of future potential trends.

**Fig. 6.**
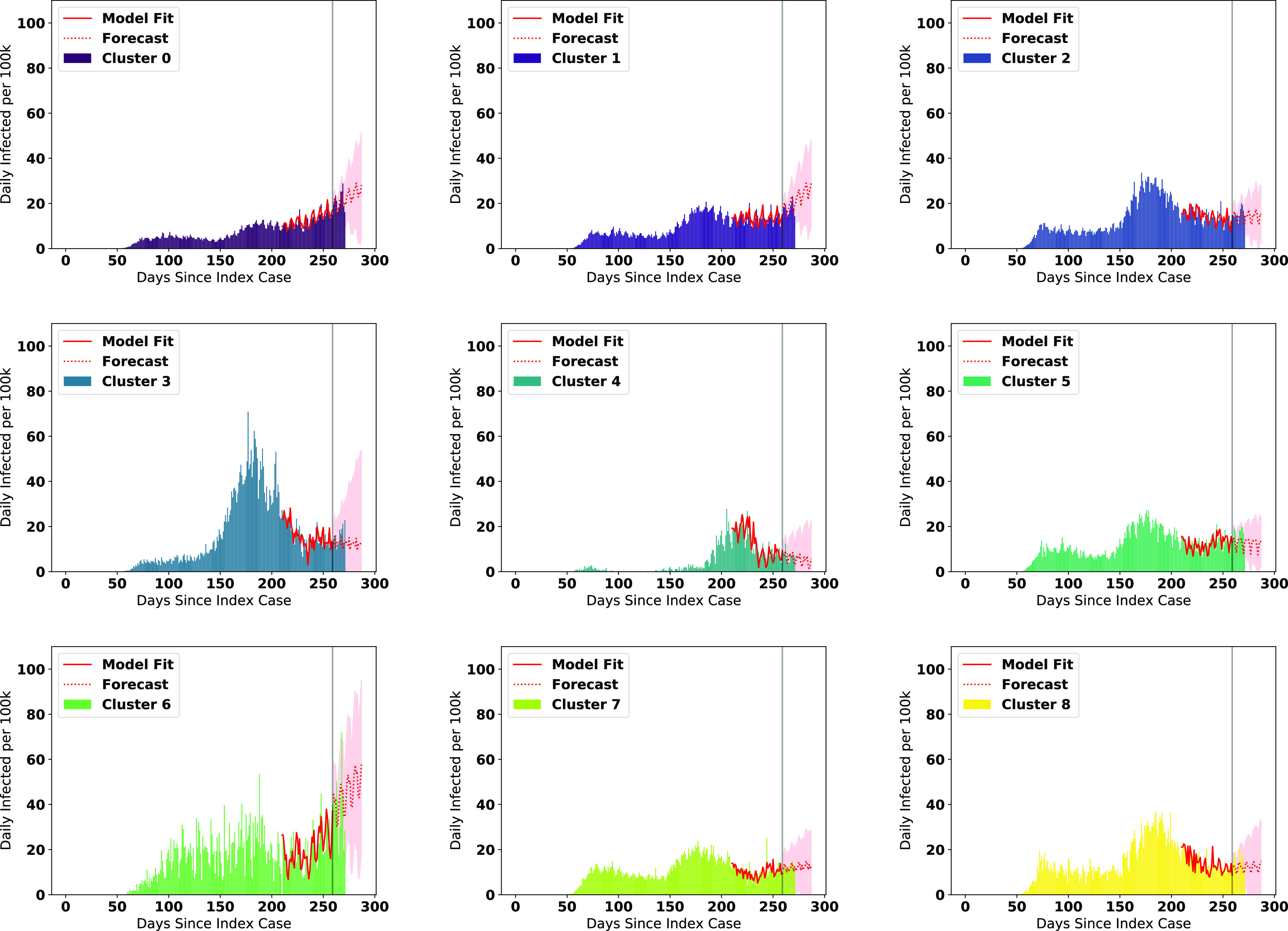
ARIMA Forecasts for each cluster (cases) during the most recent forecasting window.

**Fig. 7.**
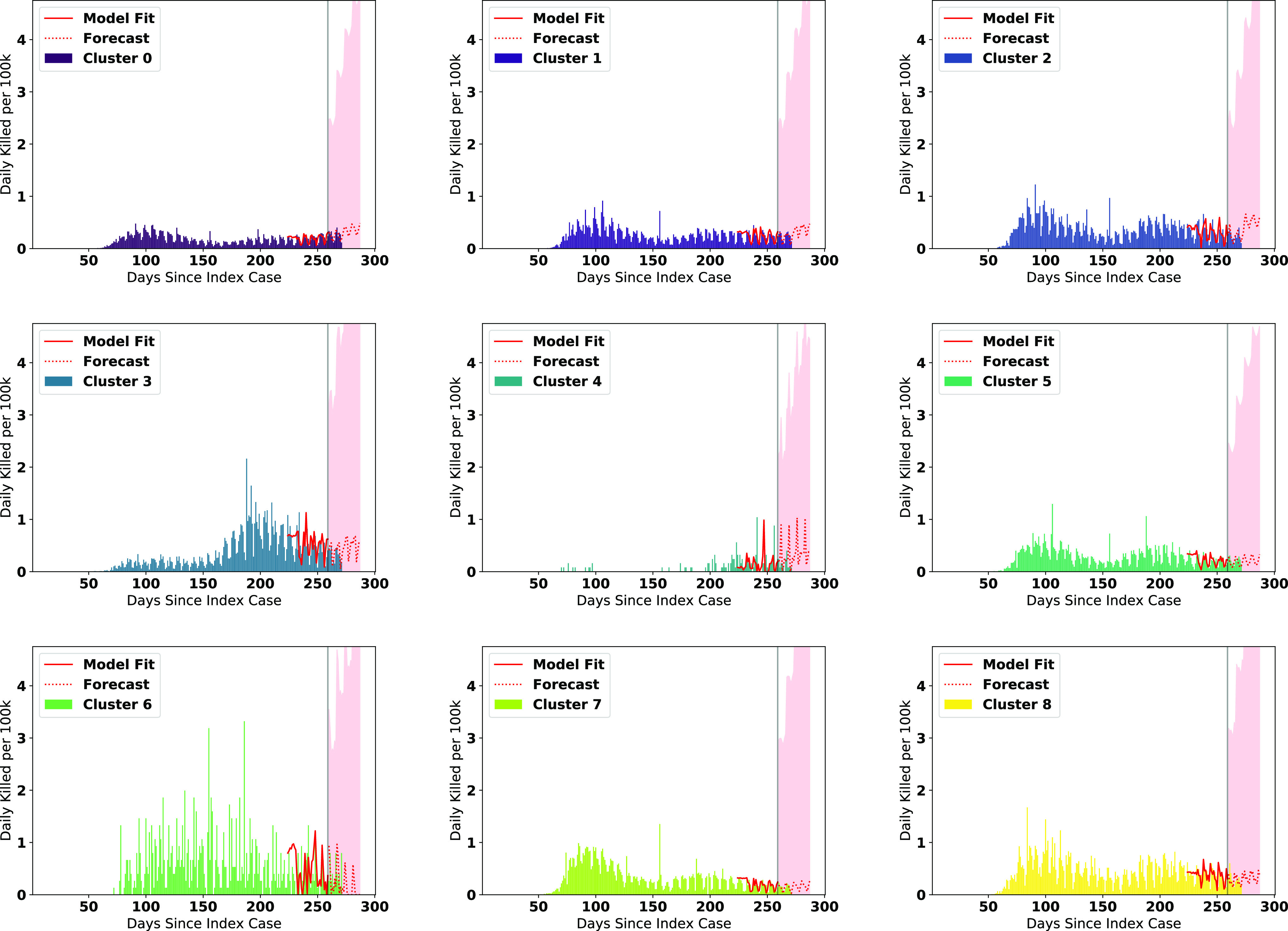
ARIMA Forecasts for each cluster (deaths) during the most recent forecasting window.

We visualize the fitted models on the data along with the forecasts for the number of infections in Fig. 6, and number of deaths in Fig. 7. Note that the figures represent the fitted/forecasted daily new active infections/deaths per 100 k people by multiplying the proportion infected/killed daily by 100,000. We observe from the figures that the last seven days of real data generally follow the trend forecasted or are within the 95% (infections)/80% (fatalities) forecast confidence intervals.

We note a few trends based on the visuals expressed in Fig. 6 and Fig. 7. The county clusters with large concentrations of minority populations (clusters 2, 3, 6, and 8) experienced disproportionately high concentrations of cases and fatalities during the first and second major infection waves, matching the findings in the literature [35], [36]. While new cases in clusters containing large Black pluralities (clusters 2 and 8) and large Latino pluralities (clusters 3 and 5) are forecasted to level off or decline heading into the holidays, we observe that in cluster 6 (large Native American pluralities), the number of new cases are forecasted to further accelerate into the holiday season. We also see that the number of new cases in White-majority county clusters (0 and 1) are expected to have some of the highest rates of acceleration of new cases heading into the holiday season.

**Fig. 8.**
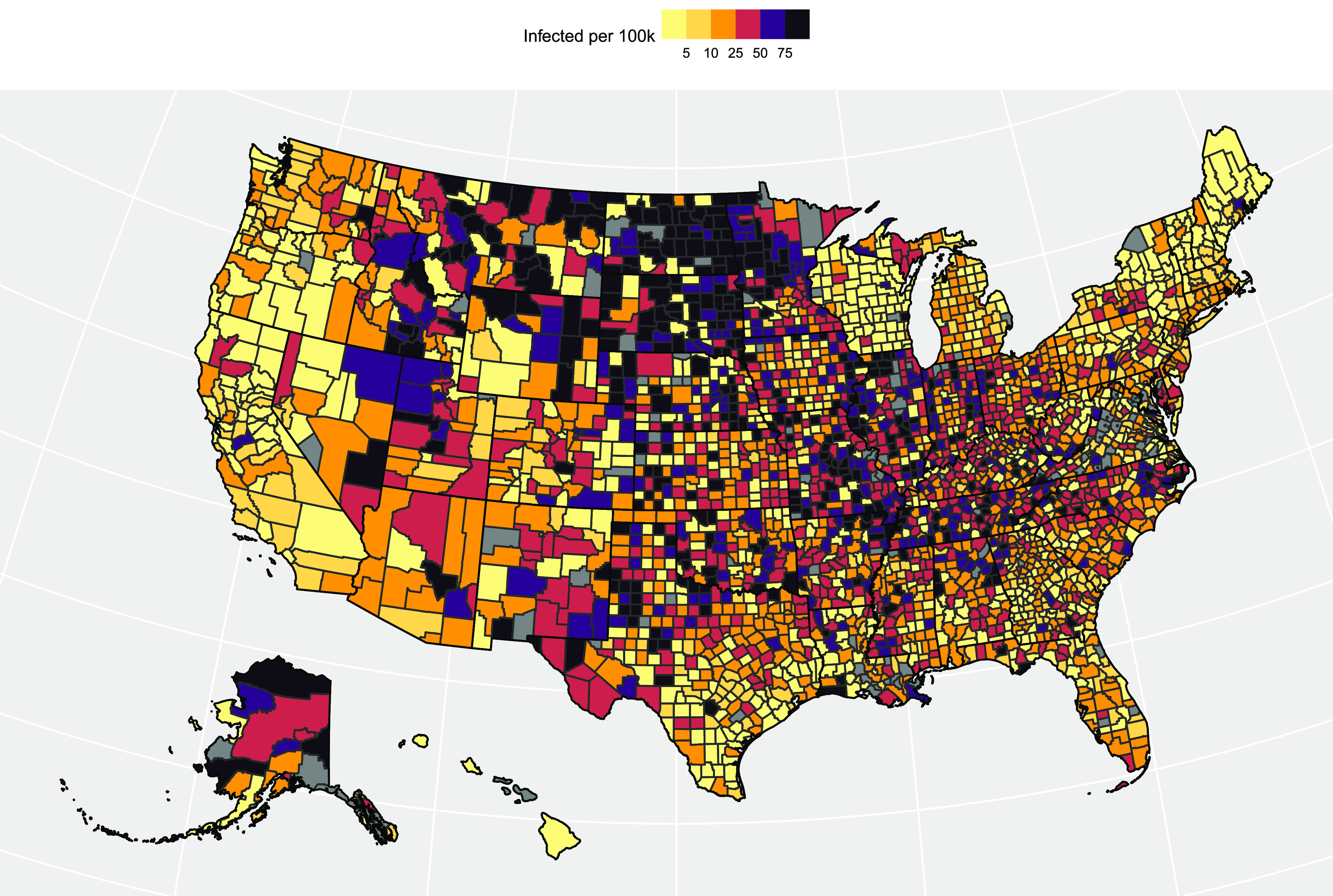
Visualizing the projected holiday season risk of new infections each county based on the clustering/time series approach. Darker counties are at higher risk (i.e., more likely to see higher cumulative proportions of their populations infected). Grey counties do not have data available.

The daily infection rates are a useful tool for determining risk. A growing trend in the daily new cases indicates an accelerating spread of SARS-CoV-2, implying the higher risk associated with the virus, whereas a flattened or decreasing trend implies containment of the virus through various means, such as wearing masks, social distancing, public health policy, etc. We gauge the risk a cluster faces by taking the average of the 22nd to 28th day forecasts (average of the 4th week out from the train/test split). These values are utilized to generate a risk heatmap for new cases and deaths (visualized in Fig. 8). From the figure, we observe that counties in the Midwestern US, particularly those in the Dakotas, are at the highest risk of increased fatalities heading into the holiday season, and are among the highest-risk counties for infection accelerations.

### B. National-Level Forecasts

**Fig. 9.**
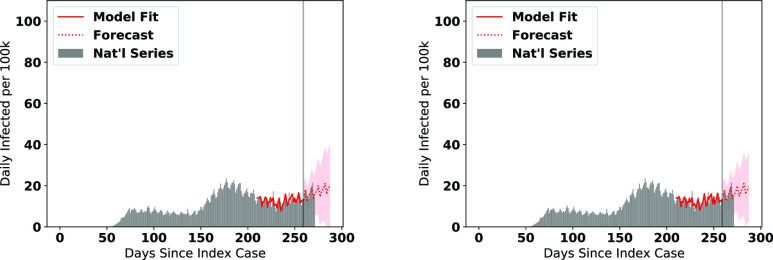
National daily active infection forecasts from pooled cluster forecasts (left) and ARIMA$(0, 1, 1)x(0, 1, 1)$ forecast (right).

**Fig. 10.**
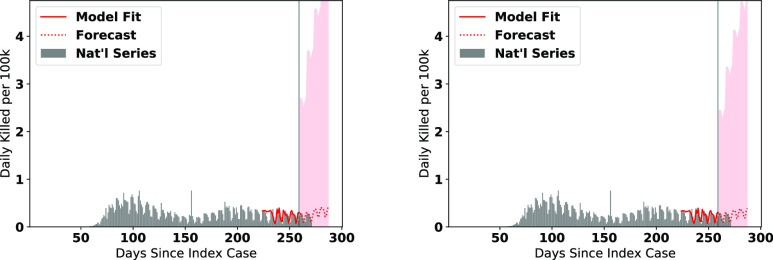
National daily fatality forecasts from pooled cluster forecasts (left) and ARIMA$(1, 0, 0)x(0, 1, 1)$ forecast (right).

Since the progression of the SARS-CoV-2 pandemic is driven predominantly in regional waves, it is worthwhile to assess how effective a national-level forecast generated by pooling together the cluster forecasts would be. To achieve the cluster aggregation forecast, we take the weighted averages of the fitted values, forecasts, and confidence intervals of all of the cluster sums. The resultant fit and forecasts for the national per-capita cases and fatalities are visualized in the left panes of Fig. 9 and Fig. 10. Upon visual inspection, the aggregated forecasts seem to fit the data well.

In order to determine a baseline for comparison, we fit an ARIMA$(0, 1, 1)x(0, 1, 1, s=7)$ model to the national daily active infections series, and an ARIMA$(1, 0, 0)x(0, 1, 1, s=7)$ model to the national fatality series (with the national cases as an exogenous factor). These orders were based on residual analysis of various fits until the ARIMA models failed to reject the null hypothesis of the Box-Ljung goodness-of-fit test. The fits of these models are visualized in the right panes of Fig. 9 and Fig. 10. In addition to the visual validation of the national-level forecast, we compute the RMSE for the ARIMA and pooled models to measure the goodness-of-fit, and the MAE/ME to measure the accuracy and bias, respectively, of the forecasts at the last forecast window only; we do not simulate the rolling forecasts as we did earlier. The results of these computations are shown in Table XIII. From the results in the table, we can conclude that both the ARIMA and aggregated models fit the data very well based on their RMSE values, and both provide high-quality forecasts. Moreover, from the visual and numerical validation, we can conclude that the cluster aggregation approach at the national level is quite effective. It fits the data very well, and provides accurate, low-bias forecasts in the validation window of one week out. Indeed, in many of the metrics, the aggregated forecast outperforms the dedicated national ARIMA model.

**TABLE XIII:** MAE, ME, and RMSE Scores of the National Forecasts. Lower (better) MAE, ME Closer to 0, and Lower RMSE Scores are in **Boldface** Font

**Metric**	**Cluster Aggregation**		**National-Only**	
	**Cases**	**Deaths**	**Cases**	**Deaths**
RMSE	$\mathbf {1.8564}$	$\mathbf {0.0756}$	1.9062	0.0839
MAE	$\mathbf {1.1143}$	$\mathbf {0.0180}$	1.1440	0.0560
ME	0.9749	$\mathbf {0.0051}$	$\mathbf {0.5848}$	0.0079

## IV. Discussion

### A. Model Risk Measurement

Since new hospitalizations, fatalities, and other costs associated with the pandemic are tied directly to the number of daily new active infections, we utilize the forecasted future daily change in infections as the main gauge of risk. The CDC has published guidelines that tie exposure risk to the average daily active cases over a 28-day period based on testing density. For example, based on a high testing density, a low exposure risk is associated with $[0, 5)$ infections per 100 k, a medium risk with $[5, 50)$ infections per 100 k, a high risk with $[50, 100)$ infections per 100 k, and an extremely high risk with >100 infections per 100 k in high-population areas [49].

After utilizing the cluster aggregation time series to fit the ARIMA models, we then applied the trained model on the individual counties normalized to a ratio along the $[0, 1]$ range by dividing the county's daily new cases/fatalities by the corresponding county population. This approach is done as a sort of “smoothing” technique; the aggregated clusters will have much less volatility in the day-to-day changes in the new active infections versus the individual counties. When the most recent ARIMA models are applied to each county (based on which cluster it is a member of), the 28-day forecasts are utilized to project the average new infections per 100 k 28 days from the last day of the model. From Fig. 8, we see that the highest-risk counties heading into the 2020 holiday season are concentrated in the Midwestern United States: The Dakotas, Missouri, Minnesota, and Illinois have the highest concentration of counties that are projected to exceed 75 new infections per 100 k over a 28-day average.

### B. Implications of Results

There are a few key takeaways from the results presented in this manuscript. First, the aggregated time series for each cluster helps highlight the inequity of the impacts of COVID-19 on minority communities at a macro level. We see from the trends that counties with large ethnic pluralities in the US experienced larger spikes during the second major transmission wave. Secondly, we see distinct geographic patterns emerge after applying the trained ARIMA models to each county. There is a concentration of higher-risk counties in the Midwestern and parts of the Southeastern US, and these counties are mostly geographically adjacent to one another. The third key insight comes with the benefit of hindsight. The model projects that the Dakotas in particular are to be extremely hard-hit by the COVID pandemic heading into the winter holiday, and this largely matched with real-world progression of the pandemic in this part of the US. Finally, it is noteworthy that when aggregated together, the combined cluster forecasts lead to a forecast that slightly outperforms a dedicated ARIMA model for the national-level data, showing that the approach presented in this paper can be applied to both county-level and with national-level decision-making. Based on this real-world success, we believe our proposed two-stage approach has promise in a DSS to aid policymakers in allocating resources in anticipation of changes in infection progression.

### C. Limitations of Current Approach

Based on the silhouette analysis of the clusters, along with the Box-Ljung test results along with the RMSE, MAE, and ME metrics for the ARIMA models, the work presented in this manuscript is a good start and may serve as a baseline to evaluate other, potentially more robust approaches. In the clustering approach, we note that there is difficulty in drawing clear demarcations between county clusters with the current set of features considered. It may be worthwhile to measure the economic interconnections between counties, as these are likely to lead to a higher likelihood of travel/contact between people living in those counties. Incorporating additional features into the clustering data may help the algorithm converge to a solution where there are more clear boundaries between the cluster sectors. For the time series models, the rejection of the null hypothesis in some time windows along with the correspondingly poor worst-case performance for a few of the scenarios shows that the ARIMA approach, while promising, can be improved upon. Improvements in the clustering approach may lead to improvement off the bat. Additional improvement may come from the exploration of long-term memory models in the time series forecasts. Two models that may be applied here are LSTM and AutoRegressive Fractionally Integrated Moving Average (ARFIMA) models, which both consider short-term trends that are most impactful on the forecast, while remembering longer-term behavior of the series overall, which may help in forecasting corner cases (the cases where our current ARIMA approach may be falling short). Normal ARIMA approaches remove the impact of long-term trends when differencing is applied, which may lead to bias in the longer-term forecasts.

### D. Next Steps for Incorporation Within a DSS

Our approach presented here can serve as an excellent baseline for building a DSS. To get a viable working DSS, the main task to address is the development of automated time series modeling that can take into account all of the data available, such as the use of LSTM and ARFIMA. These models can be compared with the results presented in this manuscript as a baseline. After the modelling approach can be automated, other steps needed to get a viable DSS would determine how to pull data on a regular basis from open repositories, how to transform the stream of data so the automated modeling approaches could leverage it, and exploring how to effectively visualize the results in a succinct way for the end-user of the DSS.

## V. Conclusion

In this paper, we developed a multi-step forecasting system, where we first cluster US counties into alike regions based on shared economic and demographic factors. We then utilized the aggregate of the case data for each region to build seasonal random walk and ARIMA forecast models. We demonstrate via Ljung-Box tests that custom ARIMA forecasting is superior, as every cluster contains either autoregressive or moving-average components, and that the forecasts are aligned with existing literature related to high-risk demographic groups and the effects of COVID-19 on their communities. Moving forward, this system has the potential to be incorporated into a Decision Support System that may aid national-level policymakers in determining how to allocate medical resources to various counties in the United States. In order to develop such a system, automatic long-term memory models should be explored in order to automate the development of the time series forecasts. Future work should also seek to improve the quality of the forecasts by incorporating several exogenous factors into the data such as more detailed health demographic data (obesity rates, cardiovascular health data, etc.), hospitalization rates related to COVID-19, population movements, etc. In addition, the national-level aggregate forecast may be improved by incorporating network analytics to define relationships between the various regional clusters that are developed based on socioeconomic factors.
